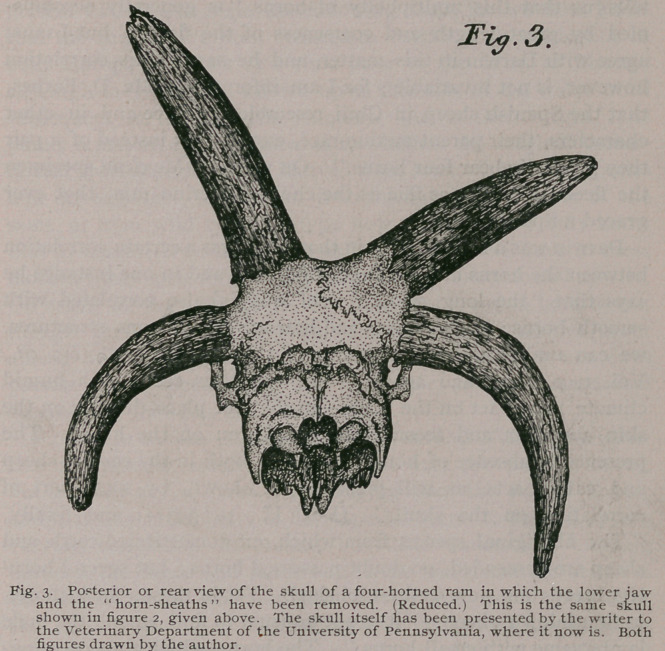# Notes on Horned Mammals, with Some Observations upon Policerate or Multiple-Horned Sheep

**Published:** 1889-01

**Authors:** R. W. Shufeldt


### Art. II.—NOTES ON HORNED MAMMALS, WITH SOME OBSERVATIONS UPON POLICERATE OR MULTIPLE-HORNED SHEEP.

BY R. W. SHUFELDT, M. D., C. M. Z. S.

So important a character are the horns in several orders of mammals that we have been enabled, in the case of the ruminants, to successfully bring them into play in the classification of the group. Sir Richard Owen, in referring to horns in general, remarks that "the weapons to which the term horn is properly or technically applied consist of very different substances, and belong to two organic systems, as distinct from each other as both are from the teeth. Thus the horns of deer consist of bone, and are processes of the frontal bone; those of the giraffe are independent bones, or 'epiphyses' covered by hairy skin; those of oxen, sheep and antelopes are 'apophyses' of the frontal bone, covered by the corium and by a sheath of true horny material; those of the prong-horned antelope consist at their bases of bony processes covered by hairy skin, and are covered by horny sheaths in the rest of their extent. They thus combine the character of those of the giraffe and the ordinary antelope, together with the expanded and branched form of the antlers of deers. Only the horns of the rhinoceros are composed wholly of horny matter, and this is disposed in longitudinal fibres, so that the horns seem rather to consist of coarse bristles compactly matted together in the form of a more or less elongated sub-compressed cone." Nowadays, so well known are those strange metamorphoses through which the deer grow and shed their antlers every year that it will not be necessary to dwell for any length upon that interesting point, suffice be it to say that the deer or Cervidæ are designated in consequence the *Solid-Horned Ruminants*. To this I may add that these antlers are grown by both sexes in the reindeer, but only by the males in all the other species; further, they *always* spring from the *frontal* bones; and finally the earliest known fossil deer were without antlers, and they slowly came into being during several geologic epochs. There are two genera of existing Cervidæ that never have antlers, *Moschus* and *Hydropotes.*

Among the bovine (*Bovidæ*) animals the horns also are supported by the *frontal* bones, and of them Professor Flower has said that they "consist of permanent conical, usually curved, bony processes, into which air-cells continued from the frontal sinuses often extend, called 'horn-cores,' ensheathed in a case of true horn, an epidermic development of fibrous structure, which grows continuously, though slowly, from the base, and wears away at the apex, but is rarely shed entire." The rarity to which the Professor alludes is our own prong-horned antelope of the Western plains, which periodically sheds its horns, an interesting process that has been most fully described by Dr. Canfield and Judge Caton of Chicago. Indeed, our antelope is an admirable go-between, for not only does it thus shed its semi-hollow horns, but when they are of full growth they are *bifurcated,* and we will add that they, too, are processes of the *frontal* bone. In all of our existing bovine ruminants the females may or may not possess horns, but the males always do. From these various circumstances the "hollow-horned" ruminants have been styled the *Cavicornia.* It was during the Miocene epoch that the geologic ancestors of these two groups of animals first began to be differentiated into "horn" and "antler-bearers," and in both cases from the *frontal* bones.

We next come, however, to a very different state of affairs, and I refer to the peculiar horns which ornament the forehead of that unique species, the giraffe. This distinguished ruminant and the only living representative of its family, has neither true horns nor true antlers, but the projections upon its forehead, more a badge of its lineage than a weapon of defence, are neither one thing or the other. They consist, however, of a pair of persistent, solid, bony appendages, which are attached, in the adult, partly to the *frontal* and partly to the adjacent *parietal* bones of the forehead. Young giraffes, when first born, already show these processes, and their development commences in separate bony nuclei, but in fully matured animals they commonly become finally attached to the skull in the manner indicated above, and are always covered by the common integument, with a brush of bristles ornamenting either of their apices. In front of these "horns" in the male giraffe there occurs another median protuberance, supported by the *frontal* and *nasal* bones, which has been spoken of as a third horn.

There is one other class of mammals that have horns on their foreheads, and these are the rhinoceroses, but in them the *nasal* bones are only roughened and thickened in order to form a proper base, but otherwise having no connection with the single or pair

*Fig. 1.*

Fig. 1. Head of four-horned Sheep from New Mexico. Drawn by the author, and much reduced.

of dense, conically-curved and burnished horns of these ponderous pachyderms. Among extinct mammals, such as the *Dinocerata*, horns of extraordinary formation were the rule, and even among our existing mammals, when we view these structures as a whole, their wonderful variety, size and fantastic forms are truly astonishing.

Darwin in his "Origin of Species" and "Animals and Plants under Domestication,'' and Spencer in his works, have pointed out with no little significance as to how in the early history of mammalian forms in geologic times, such appendages as horns may have first arisen, and we will not in the present connection enter upon a discussion of the subject here. Horns no doubt in some of the primitive types may have been nothing more than bony bosses on the *frontal* bones, and these at first only covered with the thickened skin overlying their sites. From such starting points as these, it is easy to conceive how that by a process of developmental evolution we can have in these recent times, the horns, the almost endless variety of the horns, of one existing *Bovidæ*. Other factors, too, have undoubtedly been at work, *pari passu*, to perfect other results which have eventually come to pass, as the vast array of forms in these horns, and the shedding of them in the *Cervidæ*.

I am inclined to think that the ponderous *Dinocerata* of Marsh had their frontal proturberances simply covered with hardened skin, and not encased in a hollow horn like our present *Cavicornia*.

As nicely as this may explain these paired frontal weapons, and their origin, to our mind, it in no way clears up to me the question of the growth of multiple horns. So far as I have been able to ascertain, this interesting state of affairs is only to be found among certain varieties of domesticated sheep. Quite recently, during my residence at Fort Wingate, New Mexico, it came to my knowledge that these multiple-horned sheep were to be obtained not infrequently among the extensive flocks owned by the wealthier herders among the Navajo Indians, and as these were near at hand, it was not long before I succeeded in purchasing a splendid ram, possessing two pairs of magnificent horns. I at once photographed this odd looking creature, and from the photograph made the drawing which accompanies this paper. Subsequently, I also made photographs of the skull of the same animal, and the drawings I made from these latter are shown in figures 2 and 3. This particular ram possessed a heavy coat of fine wool, that was slightly curled, while the hair upon his limbs and face was short and nearly straight. Black markings occurred on all four legs, while his entire muzzle, the ears, circum-ocular areas, and a few spots on his face, were also of the same color. The form and position of the horns can best be appreciated by a glance at figure 1, where it will be seen that the lower pair somewhat interfered with the mobility of the animal's ears, but this is not an uncommon occurrence even among the true Merino sheep. He was considerably larger, however, than the average domesticated ram, standing up nearly as tall as a Bighorn Sheep of the Rocky Mountains. It will be observed, too, from the figure that the apices of three of his horns are either broken off or were intentionally cut off, while his face showed some deep scars, received, no doubt, in conflicts with other rams of the herd.

*Fig. 2.*

Fig. 2. Right lateral view of the skull of a four-horned sheep (reduced). The "horn-sheaths" have all been removed.

Prior to the year 1865 the Navajo Indians did not own many, if any sheep, but notwithstanding this fact, all my efforts to ascertain from them, and from the white interpreters among them, where these multiple-horned sheep were originally obtained, failed. They say they have owned the stock for many, many years. Questioning them in reference to the highest number of horns they have ever observed in any single ram, elicited the information, that the horns are never possessed except by the males, and that there may be three, four, five, or six of them, but never any more, and that they are shown in the kids very soon after birth.

It will be seen from my drawings of the skull of this sheep, that all these horns spring from the *frontal* bones of the cranium, and in no instance encroach upon the parietals or much less, the nasals. Now Darwin says in vol. I of his "Animals and Plants under Domestication," that these "horns, when numerous arise

*Fig. 3.*

Fig. 3. Posterior or rear view of the skull of a four-horned ram in which the lower jaw and the "horn-sheaths" have been removed. (Reduced.) This is the same skull shown in figure 2, given above. The skull itself has been presented by the writer to the Veterinary Department of the University of Pennsylvania, where it now is. Both figures drawn by the author.

from a crest on the frontal bone, which is elevated in a peculiar manner." (p. 120); and I may add that in the same connection he speaks of specimens wherein he has seen as high as eight well-developed horns on the head of the same ram. This is an interesting case of the *force of heredity*, for the frontal bones are even distorted to accommodate this multiplicity of appendages, which latter, in sheep, never encroach upon the adjacent bones; and even were they to do so they would in that particular only agree with such a species as the giraffe. How stubborn is nature in some of her performances!

According to other authorities before me, these policerate sheep are also to be found in Southern Russia, and another seems to think that "the influence of climate is remarkably shown in the tendency of the Merino breed to develop an additional pair of horns, when transferred from Spain to Peru," (Jenks). Youatt tells us that this multiplicity of horns "is generally accompanied by great length and coarseness of the fleece," but I must agree with Darwin in this matter, and he says "this correlation however, is not invariable; for I am informed by Mr. D. Forbes, that the Spanish sheep in Chili resemble, in fleece and in other characters, their parent merino-race, except that instead of a pair they generally bear four horns." On my New Mexican specimen the fleece was fully as fine as the choicest Merino ram, that ever graced a Spanish pasture.

Darwin was a firm believer in that there was a certain correlation between the horns and the fleece of sheep, and in one instance he says that "the long, smooth wool was [is] also correlated with smooth horns; and as horns and hair are homologous structures, we can understand the meaning of this correlation," (*loc. cit.*, Vol. I, p. 127), and again, "We can thus see how a humid climate might act on the horns—in the first place directly on the skin and hair, and secondly by correlation on the horns. The presence or absence of horns, moreover, both in the case of sheep and cattle, acts, as will presently be shown, by some sort of correlation on the skull." (Vol. II., p. 393)[1]; and finally, "The aboriginal species from which our domesticated cattle and sheep are descended, no doubt possessed horns; but several hornless breeds are now well established. Yet in these—for instance, in Southdown sheep—"it is not unusual to find among the male lambs some with small horns." The horns, which thus occasionally reappear in other polled breeds, either "grow to the full size," or are curiously attached to the skin alone and hang "loosely down, or drop off" [Youatt]. The Galloways and Suffolk cattle have been hornless for the last one hundred or one hundred and fifty years, but a horned calf, with the horn often loosely attached, is occasionally born" (*loc. cit.*, Vol. II, p. 43).

---

1. With reference to this latter point, he further on in the same work says, "With our cattle and sheep the horns stand in close connection with the size of the skull, and with the shape of the frontal bones; thus Cline found that the skull of a horned ram weighed five times as much as that of a hornless ram of the same age." "*Animals and Plants under Domestication.*" (*Vol. II., p. 401*).

Turning to Youatt, he says in respect to horned sheep, that those with a multiplicity of horns never are found "in any breed of much value;" while Sturm states that in the various breeds, the curlier the wool, the more the horns are spirally twisted.

In concluding then, we are enabled, so far as our knowledge at present extends, to state the following facts, for we now know that the sheep have been reduced to a state of domestication in both Europe and Asia at a time prior to the dawn of history, although they were not known upon this side of the water until subsequent to the Spanish conquest. As to their origin, it is purely a matter of conjecture, as to whether the original domesticated species arose or was derived from some of the wild species now in existence, or from an interbreeding of a number of the same, or from wild types or type now extinct. We only know that by careful crossing of the skilled breeders we have in these modern times some forty or fifty well-defined domesticated species. On nearly all the wild species the horns are found in both sexes, though smaller in the females, while hornless varieties occur among the domesticated forms, where neither rams nor ewes possess these appendages; and as a rule, in the domesticated species only the rams have horns.

Finally, in various parts of the world, several of the tame varieties may possess, usually the rams, not only two pairs of horns, but as many as *four* pairs, all springing from the frontal bones; and that this multiplicity of development seems to depend upon a varying correlation, at present but little understood, existing between the fleece and horns on the one hand, and the action of the climate on the other,—which, if it be true, would go to show that this interesting correlation between such homologous structures as the wool and the horns, may be so exacting, as when set in operation by such a factor as a profound change of climate, reacts upon so a deep structure as the skull, as to force its frontal bones to develop the necessary bony cores to support the horns. Not only this, but the correlation appears further to be of such a nature, that when the horns are markedly spiral in form, the wool is eminently curly in character. Strange, indeed, sometimes is the influence of climatal environment upon organized structures, and it is but fair to confess that we are as yet but a long ways from a full comprehension of such interactions, in the vast majority of instances.

## Figures and Tables

**Fig. 1. f1:**
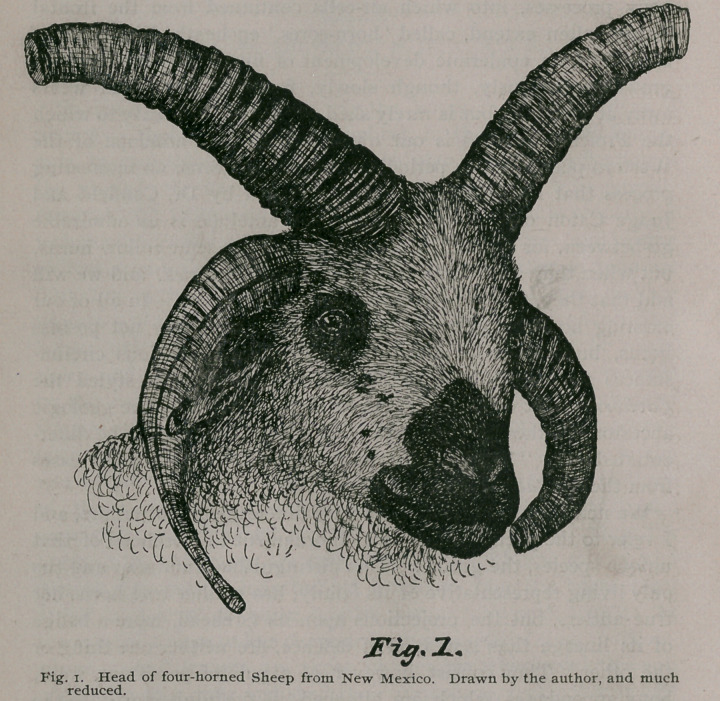


**Fig. 2. f2:**
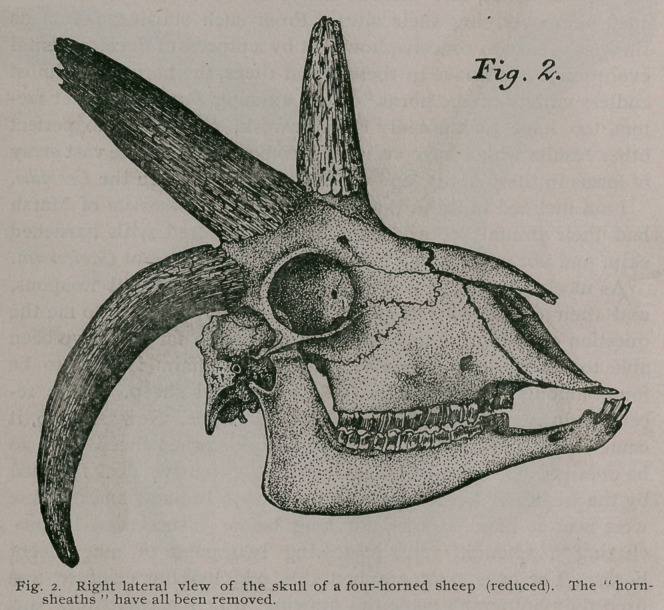


**Fig. 3. f3:**